# Small intestinal mucosal abnormalities using video capsule endoscopy in intestinal lymphangiectasia

**DOI:** 10.1186/s13023-023-02914-z

**Published:** 2023-10-02

**Authors:** Lin Lin, Kuiliang Liu, Hong Liu, Jianfeng Xin, Yuguang Sun, Song Xia, Wenbin Shen, Jing Wu

**Affiliations:** 1 Department of Gastroenterology, Beijing Shijitan Hospital, Capital Medical University, Haidian District, Beijing, China; 2 Department of Lymphatic Surgery, Beijing Shijitan Hospital, Capital Medical University, Haidian District, Beijing, China; 3 Clinical Center for Lymphatic Disorders, Capital Medical University, Beijing, China; 4 Department of Gastroenterology, Beijing Friendship Hospital, Capital Medical University, Beijing, China

**Keywords:** Intestinal lymphangiectasia, Video capsule endoscopy, Complication, Small intestine

## Abstract

**Background:**

Intestinal lymphangiectasia (IL) is a rare protein-losing enteropathy caused by disorders of the intestinal lymphatics. There are only a few case reports and case series concerning the VCE (video capsule endoscopy) findings of IL. This work aimed to evaluate the VCE characteristics of small intestinal mucosal abnormalities in patients with IL, and to investigate the relationship between clinical and VCE characteristics.

**Methods:**

Consecutive patients with IL who underwent VCE were enrolled in this retrospective study. The cases were classified into the white villi group and non-white villi group according to mucosal abnormalities detected by VCE. Clinical and endoscopic characteristics were investigated and analyzed.

**Results:**

A total of 98 patients with IL with a median onset age of 26.3 ± 19.2 years were included. VCE revealed the following small intestinal lesions: (i) white villi type (57/98, 58.2%), i.e.: white-tipped or granular villi, white nodular villi or plaques; (ii) non-white villi type (41/98, 41.8%), i.e.: diffused low and round villi; (iii) complications (46/98, 46.9%), i.e.: bleeding, ulcers, protruding or vesicular-shaped lesions, stenosis and lymphatic leakage. A total of 58.2% (57) and 41.8% (41) of the cases were classified into the white villi and non-white villi groups respectively. The percentage of chylothorax in the white villi group was significantly lower than that in the non-white villi group (12/57 vs. 19/41, *p* = 0.008). In VCE, there were no significant differences in the involved segments and total detected rate of complications between the white villi and non-white villi groups (*p* > 0.05), while the detected rate of lymphatic leakage in the white villi group was significantly higher than that in the non-white villi group (31.6% vs. 12.2%, *p* = 0.026).

**Conclusions:**

Our study evaluated the entire small intestinal mucosal abnormalities of IL by VCE, especially endoscopic complications. IL has specific VCE abnormalities in addition to classical endoscopic findings.

## Background

Intestinal lymphangiectasia (IL) is a rare protein-losing enteropathy (PLE) caused by disorders of the intestinal lymphatics [1]. It is characterized by the blockage of lymph fluid draining from the small intestine [2, 3]. As lymphatic fluid contains a large amount of protein, fat and lymphocytes, leakage will cause hypoproteinemia, lymphocytopenia, and decreased levels of immunoglobulin in serum. The common clinical manifestations of IL include hypoproteinemia, bilateral lower limb edema, and ascites [4].

According to the etiology, IL can be classified into primary and secondary IL. Primary intestinal lymphangiectasia (PIL) is considered a primary congenital disorder [5], whose prevalence is unknown, and the disease distribution around the world is poorly investigated. The precise mechanism by which IL develops remains unclear. Although several hereditary factors, including vascular endothelial growth factor receptor 3, C, and D genes may be involved in the pathogenesis of PIL, almost all the reported cases are of sporadic origin [6]. Secondary intestinal lymphangiectasia (SIL) is always secondary to a disease blocking intestinal lymph drainage. This includes extensive abdominal or retroperitoneal carcinoma or lymphoma, chronic pancreatitis, retroperitoneal fibrosis, mesenteric tuberculosis or sarcoidosis, constrictive pericarditis, and chronic heart failure [4, 7].

Endoscopy is one of the main modalities for the diagnosis of IL. However, conventional esophagogastroduodenoscopy (EGD) and colonoscopy cannot visualize the entire intestine. Single balloon or double balloon enteroscopy is invasive and cannot ensure the complete evaluation of the entire small intestine. Video capsule endoscopy (VCE), a novel device introduced in the last decade, could be used to explore the whole segment of the small intestine [8, 9] and is a suitable option for the evaluation of IL [4, 10].

To the best of our knowledge, there are only a few case reports and case series concerning the VCE findings of IL [10–15]. In this study, the aim was to evaluate the VCE characteristics of small intestinal mucosal abnormalities in patients with IL, and to investigate the relationship between clinical and VCE characteristics.

## Methods

### Patients and clinical data

This retrospective study was implemented at the Digestive Endoscopy Center and Department of Lymphatic Surgery, Beijing Shijitan Hospital, Capital Medical University, China, from January 2011 to December 2021. Consecutive patients with IL who underwent VCE were enrolled in the study. Patients with pacemakers, pregnant, breastfeeding, those with incomplete clinical data or lost to follow-up were excluded.

Data from the following variables was collected: age, sex, main complaint, medical history, biochemical test, radiological results, VCE findings and treatment.

All patients or legal guardians of the patients gave informed consent before VCE. This study was approved by the Research Ethics Committee of the Beijing Shijitan Hospital, Capital Medical University (sjtkyll-lx-2022074). All the data were anonymously collected and analyzed.

### Diagnosis of IL

The diagnosis of IL was suspected in patients with the evidence of PLE and confirmed by the presence of typical endoscopic findings or pathologically confirmed dilation of lymphatic lacteals in endoscopic biopsy or surgical specimens. PLE was confirmed by technetium-99 m human serum albumin (HSA) scintigraphy, accompanied by hypoproteinemia (serum albumin value < 30 g/L) [16–18].

According to the etiology, IL was classified into PIL and SIL [5].

### VCE examination protocol and image review

VCE (Pillcam SB1/SB2/SB3; Given Imaging, Yogneam, Israel) was used. All patients followed a clear liquid diet for 24 h with fasting for 12 h prior to the examination. Polyethylene glycol (PEG, 4000 ml separately) or oral electrolyte solution (3000 ml, for patients younger than 10 years old) was used for the bowel preparation. Fasting patients swallowed a capsule with a glass of water, 2 h after which they were allowed to drink or eat. VCE recording lasted 8–16 h. Video data stored on a portable device was then transferred to a computer to be analyzed by RAPID Reader® 7/8 software (Given Imaging). Images were displayed with two windows at 10 times speed in manual mode and reviewed by two endoscopists independently (Lin Lin and Kuiliang Liu). The consensus was reached after discussion if the opinion was not consistent. During the review, the entire small intestine was divided into three equal parts (proximal 1/3, middle 1/3, and distal 1/3) according to the transit interval of the capsule in the small intestine and then evaluated individually according to the method described by Goldstein et al. and Gralnek et al. [19].

### Endoscopic classification of IL

**Fig. 1:**
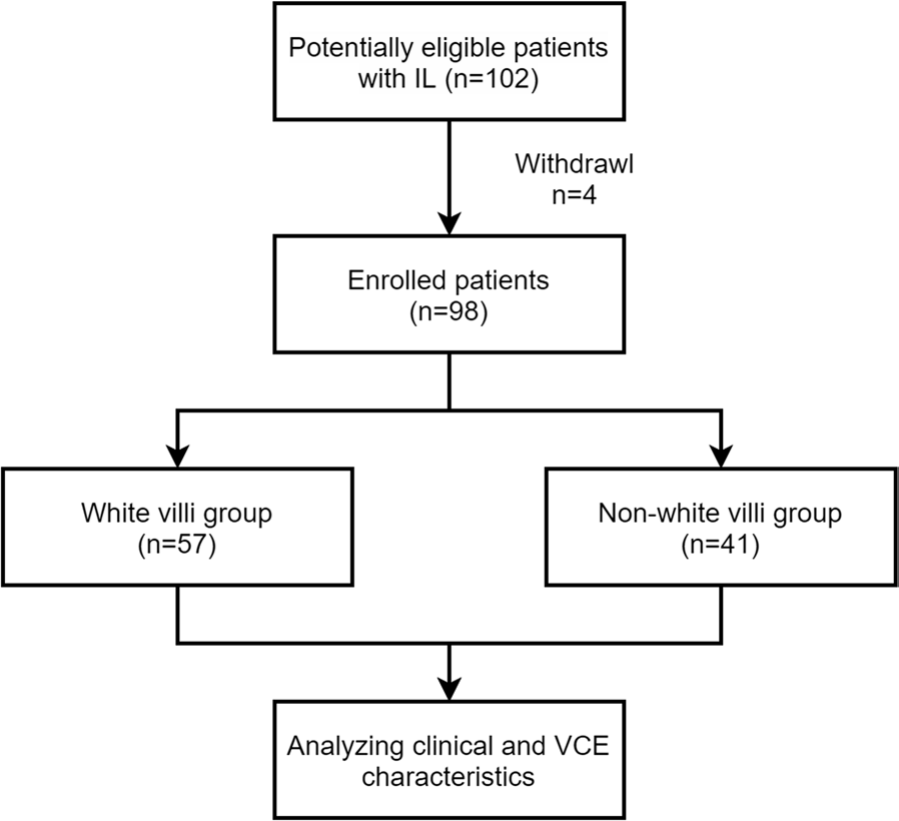
Flow diagram showing the different groups of patients with intestinal lymphangiectasia. IL, intestinal lymphangiectasia; VCE, video capsule endoscopy

In this study, the cases were classified into the white villi group and non-white villi group according to mucosal abnormalities detected by VCE. A flow diagram showing the different groups of patients with IL is described in Fig. 1.

According to Ohmiya N et al., endoscopic mucosal abnormalities were classified into the white villi type and non-white villi type [16]. The white villi type (typical lymphangiectasia) was defined as white plaques and white-tipped villi that were scattered in the small intestine. The non-white villi type was defined as apparently normal but under more detailed observation, low and round villi with a normal color that were diffused in the small intestine.

Complications detected by VCE were recorded as bleeding, ulcers, protruding or vesicular-shaped lesions, stenosis and lymphatic leakage. Lymphatic leakage was defined as whitish chyle detected in the intestinal lumen.

### Statistical analysis

The data was analyzed using Statistical Product and Service Solution version 23.0 (SPSS, Inc., Chicago, IL, USA). Categorical variables were expressed as numbers and percentages (%) and compared using the chi-square (χ^2^) test or Fisher’s exact test. Continuous variables were expressed as the means ± standard deviation (SD) and range and compared using the Student’s t test. All p values were two-tailed and the level of statistical significance was set at 0.05.

## Results

### Basic clinical characteristics

A total of 98 patients, including 53 males (54.1%) and 45 females (45.9%), with a median age at diagnosis of 32.0 ± 18.7 years (range: 4–72 years) and median age at onset of 26.3 ± 19.2 years (range: 0–68 years) were included. The median duration of disease from the onset of symptoms to diagnosis was 5.7 ± 6.8 years (range: 0.1–44 years). The onset age of 35 patients (35.7%) was lower than 18 years.

Among them, 68 patients (69.4%) were diagnosed with PIL, and 30 patients (30.6%) were diagnosed with SIL. Secondary epidemic factors include portal hypertension (n = 15), chronic heart failure (n = 7), systemic lupus erythematosus (n = 2), thrombophilia (n = 4), and cryptogenic multifocal ulcerous stenosing enteritis (n = 2). The main complaints at onset included edema (79/98, 80.6%), diarrhea (55/98, 56.1%), abdominal distension (53/98, 54.1%), fatigue (52/98, 53.1%) and abdominal pain (11/98, 11.2%). In addition, 31 patients (31.6%) suffered from chylothorax, and 52 patients (53.1%) suffered from chylous ascites.

All patients were advised for low-fat diet supplemented with medium chain triglycerides (MCT). Thoracic duct surgery to reduce lymphatic pressure was performed on 86 patients (87.8%); among them, other surgical methods, such as exploratory laparotomy and partial enterectomy, were performed on 15 patients.

### Imaging characteristics of IL in VCE

**Fig. 2:**
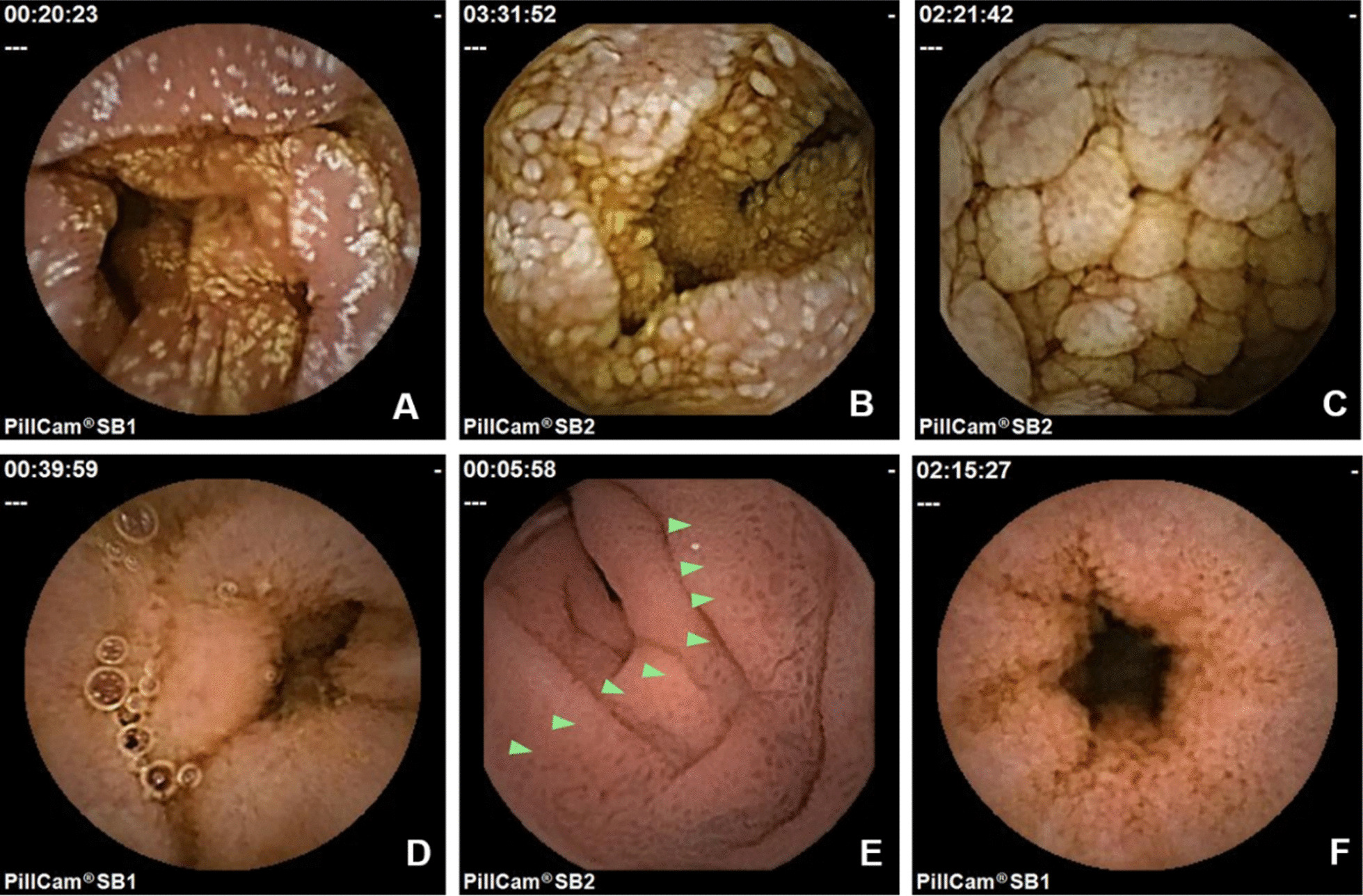
Video capsule endoscopic characteristics of intestinal lymphangiectasia. **A**. white-tipped or granular villi; **B**-**C**. white nodular villi or plaques; **D**-**F**. diffused low and round villi with edema

**Fig. 3:**
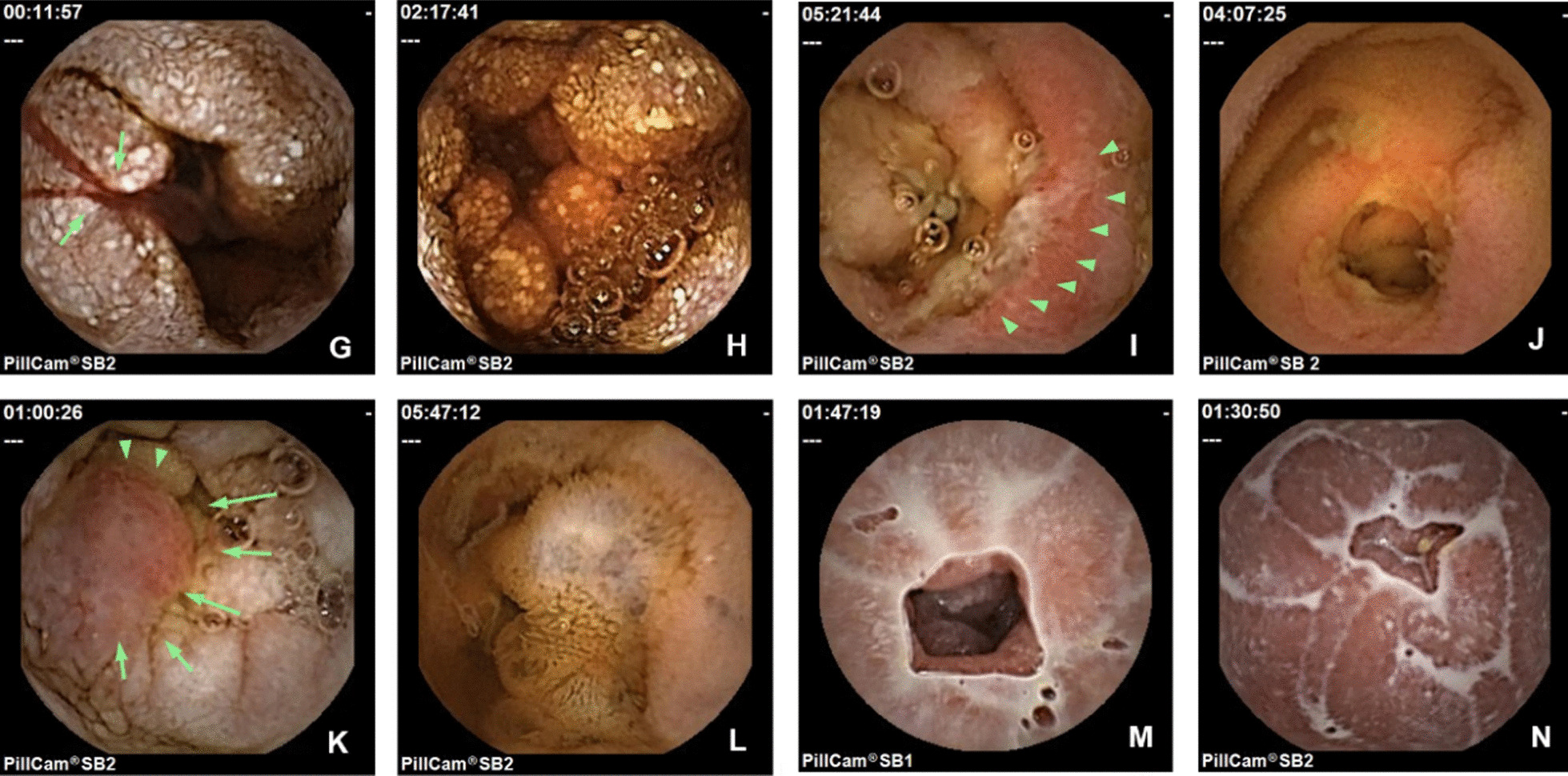
Typical complications of intestinal lymphangiectasia in video capsule endoscopy. **G**-**H**. bleeding with white-tipped or granular villi; **I**. ulcer; **J**. ulcer with significant stenosis; **K**. red protruding lesion; **L**. multiple blue vesicular-shaped lesions; **M**–**N**. lymphatic leakage

VCE revealed the following small intestinal lesions: (i) white villi type (57/98, 58.2%), i.e.: white-tipped or granular villi, white nodular villi or plaques (Fig. 2 A-C); (ii) non-white villi type (41/98, 41.8%), i.e.: diffused low and round villi (Fig. 2 D-E); (iii) complications (46/98, 46.9%), i.e.: bleeding, ulcers, protruding or vesicular-shaped lesions, stenosis and lymphatic leakage (Fig. 3).

A total of 96.9% (95/98), 65.3% (64/98) and 49% (48/98) of the cases had the small intestinal abnormalities involving the proximal 1/3, middle 1/3, and distal 1/3 segments, respectively.

### Comparison of clinical manifestations between the white villi and non-white villi groups

As shown in Table 1, in all patients with IL, 58.2% (57) and 41.8% (41) of the cases were classified into the white villi group and non-white villi group respectively. The mean onset age of patients in the white villi group was significantly lower than that in the non-white villi group (21.8 ± 17.3 years vs. 32.4 ± 20.2 years, *p* = 0.006), while the mean duration before diagnosis in the white villi group was significantly longer than that in the non-white villi group (7.0 ± 7.7 years vs. 3.9 ± 4.9 years, *p* = 0.025). More cases of SIL were detected in the non-white villi group than that in the white villi group (19/41 vs. 11/57, *p* = 0.004). The percentage of chylothorax in the white villi group was significantly lower than that in the non-white villi group (12/57 vs. 19/41, *p* = 0.008). The levels of triglyceride and immunoglobulin G in the white villi group were significantly higher than those in the non-white villi group, while the level of complement 3 was significantly lower (*p* < 0.05). There were no significant differences in main complaints, chylous ascites, the levels of lymphocytes, hemoglobin, serum albumin, serum globulin, or C-reactive protein (*p* > 0.05).

**Table 1:** Comparison of clinical manifestations between the white villi and non-white villi groups

Variables	White villi group (n = 57)	Non-white villi group (n = 41)	P
Male gender (%)	31 (53.4)	22 (53.7)	0.943
Onset age, Years, X ± SD (range)	21.8 ± 17.3 (0–59)	32.4 ± 20.2 (2–68)	0.006
Duration time before definite diagnosis, years, X ± SD (range)	7.0 ± 7.7 (0.1–44)	3.9 ± 4.9 (0.1–23)	0.025
Diagnosis Age, years, X ± SD (range)	28.8 ± 17.9 (4–65)	36.3 ± 19.0 (5–72)	0.048
Etiology			0.004
Primary (%)	46 (80.7)	22 (53.7)	
Secondary (%)	11 (19.3)	19 (46.3)	
Main complaints			
Edema (%)	45 (78.9)	34 (82.9)	0.623
Diarrhea (%)	36 (63.2)	19 (46.3)	0.098
Abdominal distension (%)	34 (59.6)	19 (46.3)	0.192
Fatigue (%)	32 (56.1)	20 (48.8)	0.471
Abdominal pain (%)	8 (14.0)	3 (7.3)	0.299
Chylothorax (%)	12 (21.1)	19 (46.3)	0.008
Chylous ascites (%)	30 (52.6)	22 (53.7)	0.920
Laboratory test			
Lymphocyte (× 10^9^/L)	1.6 ± 2.5	1.3 ± 1.2	0.570
Hemoglobin (g/L)	134.1 ± 24.3	132.3 ± 27.7	0.733
Serum albumin (g/L)	21.8 ± 6.3	22.1 ± 5.4	0.812
Serum globulin (g/L)	17.6 ± 6.6	16.0 ± 4.8	0.188
Triglyceride (mmol/L)	1.0 ± 1.1	1.7 ± 1.2	0.002
Immunoglobulin G (g/L)	4.0 ± 2.0	5.7 ± 5.2	0.045
Complement 3 (g/L)	1.0 ± 0.3	0.9 ± 0.3	0.026
C-reactive protein (mg/L)	5.6 ± 9.8	6.2 ± 9.6	0.778
Thoracic duct surgery (%)	53 (93.0)	33 (80.5)	0.063
Combined with other surgical methods (%)	7 (13.2)	8 (24.2)	0.190

### Small intestinal abnormalities between the white villi and non-white villi groups

As shown in Table 2, there were no significant differences in the involved segments between the white villi and non-white villi groups (p > 0.05). In both groups, the proximal 1/3 segment was the most frequently involved segment of the small intestine, while the distal 1/3 segment was the least involved segment.

**Table 2:** Comparison of small intestinal abnormalities between the white villi and non-white villi groups on video capsule endoscopy

Variables	White villi group (n = 57)	Non-white villi group (n = 41)	p
Involved segments of the small intestine			
Proximal 1/3 (%)	57 (100)	38 (92.7)	0.038
Middle 1/3 (%)	36 (63.2)	28 (68.3)	0.598
Distal 1/3 (%)	25 (43.9)	23 (56.1)	0.232
Complications			
Bleeding (%)	6 (10.5)	5 (12.2)	0.796
Ulcers (%)	3 (5.3)	7 (17.1)	0.089*
Protruding / vesicular-Shaped lesions (%)	6 (10.5)	6 (14.6)	0.541
Stenosis (%)	0 (0)	2 (4.9)	0.173*
Lymphatic Leakage (%)	18 (31.6)	5 (12.2)	0.026
Total (%)	28 (49.1)	18 (43.9)	0.609

There was no significant difference in the total detected rate of complications in the different groups (*p* > 0.05), but the detected rate of lymphatic leakage in the white villi group was significantly higher than that in the non-white villi group (31.6% vs. 12.2%, *p* = 0.026).

## Discussion

A total of 98 cases of IL were reviewed in our study. The median onset age was 26.3 ± 19.2 years (range: 0–68 years). The onset age of 35 patients (35.7%) was lower than 18 years. Before the definite diagnosis, the duration time varied. A total of 69.4% of the cases were diagnosed as PIL, and secondary etiologies were found in 30.6% of the cases. PIL is considered congenital in origin, most likely caused by failure in the proper formation of lymphatic vessels during infancy or earlier, and mutation or dysfunction of some genes or transcriptional factors, such as vascular endothelial growth factor-C, VEGFR3, prosperorelated homeobox-transcriptional factor, forkhead transcriptional factor and SOX18, have been reported to be related to lymphedematous diseases and lymphangiectasia [20–22]. IL can also be a consequence of acquired obstruction of the lymphatic system or age-related lymphatic changes [23]. IL may also be diagnosed in adults, even in elderly patients [16]. The onset age of IL is variable. It is suggested that the classical syndrome of clinical manifestations is only one end of the clinical spectrum of IL and a subset of IL might have a benign prognosis and therefore might be diagnosed late in life [12, 24, 25].

Typical findings of dilated lymphatics, such as pinhead-sized, white-yellow lesions can usually be detected in the second part of the duodenum by EGD in some IL cases [4, 26]. Abnormalities in the entire small intestine are always ignored. In some cases, it is possible that typical findings could not be visible in EGD and random biopsies might be negative [4, 27]. In a systematic review [4], the missing rate of routine endoscopy diagnosis was 14% (9/64).

The present endoscopic classifications of IL were always based on EGD, colonoscopy, intraoperative enteroscopy and via enteroscopy [16, 28]. A full inspection of the small intestine is necessary to confirm the diagnosis. Whether the current endoscopic classification of IL applies to the entire small intestine is unknown, and the presence of small intestinal lesions has also not been sufficiently evaluated [4, 7]. IL can vary widely in extent, manifestations, and severity [2]. Although whitish spots or plaques are commonly considered as typical findings of IL [4], pathologically confirmed lymphangiectasia can also occur without whitish lesions. In our study, entire small intestinal abnormalities, with a total of 98 cases, were evaluated by VCE. Similar to previous studies, diffuse white villi changes were important and typical lesions. There were no white villi changes in the small intestine in 41 cases (41.8%) of IL.

In addition, some complications were described by VCE, such as bleeding, ulcers, protruding or vesicular-shaped lesions, stenosis and lymphatic leakage. Nodular protrusion without whitish mucosa has been described as one kind of endoscopic finding of IL [29, 30]. In our study, similar protruding lesions were detected in both white villi and non-white villi cases. Different endoscopic characteristics may correspond to the presence of lymphangiectasia in the superficial lamina propria and deep subserosa or mesentery [16]. Ohmiya et al. [16] suggested that patients presenting as the non-white villi subtype had higher serum immunoglobulin levels and better responses to corticosteroids, which is consistent with the same trend in this study.

One of the major complications of IL is lymphatic leakage causing hypoproteinemia and malnutrition. Traditionally, 99mTc-DX lymphatic imaging and direct lymphangiography can be used to detect lymphatic leakage according to the presence of radioactivity leaking out of lymphatics and into the intestine [17, 31]. Sometimes, intraoperative endoscopy can be used to judge the location of lymphatic fluid leakage with the assistance of methylene blue [18]. One of the advantages of VCE is to localize the whitish fluid in the intestinal lumen directly as a preoperative examination. In our study, lymphatic fluid leaking into the intestinal lumen was detected in 23 cases (23.5%). Preoperative VCE detected the presence of chylous leakage, suggesting that VCE had positive value in the location of lymphatic leakage. The percentage of lymphatic leakage in the white villi group was significantly higher than that in the non-white villi group (31.6% vs. 12.2%). Bleeding is another complication of IL [32]. Bleeding lymphangiectasia may be caused by the increased intraluminal pressure causing the opening of latent lymphatic-venous connections and retrograde flow of blood into the lymphatics and bursting of blood-filled dilated lymphatics [33, 34]. There was no significant difference in bleeding between groups in our study. In our study, other complications, such as ulcers, protruding or vesicular-shaped lesions and stenosis were described. Protruding or vesicular-shaped lesions have ever been described as another type of the same class with white villi type (including nodular type and granular type) in one previous study [28]. However, in our study, protruding or vesicular-shaped lesions were detected in both the white villi and non-white villi groups. We think it is more reasonable to classify these lesions as a kind of complication of IL. Although there have been previous reports of PIL causing intussusception and intestinal obstruction [35, 36], more investigation is required to determine the relationship between IL and intestinal stenosis.

Nutritional therapy comprising a high-protein, low-fat diet, supplemented with MCT is the simplest, most effective, and widely prescribed treatment with the fewest side effects [37, 38]. Octreotide or sirolimus is also a treatment of choice in conservative therapy when MCT failed [39–41]. When conservative treatment is ineffective, lymphatic surgery including direct lymphangiography ( with computerized tomography lymphangiography after that) and thoracic duct surgery, which is believed to detect the structural abnormality of lymphatic circulation and reduce the circulating pressure, will be employed in our center [18, 39]. Laparotomy with local intestinal resection is preserved for unresponsive cases with segmental and localized lesions [42, 43]. In our study, in addition to nutritional therapy, most of the patients (86/98) underwent the thoracic duct surgery, meanwhile several patients underwent other surgical methods.

This study summarized the clinical and endoscopic characteristics of IL, and highlighted the usefulness of VCE in the entire small intestinal mucosal abnormalities. Although IL is a rare disease, a total of 98 cases were enrolled in our study, the largest number using VCE to assess IL to date. It is ideal if the appropriate strategy of treatments and surveillance could be formulated according to the endoscopic classification. More investigation is required in the future to evaluate the clinical relationship of the endoscopic abnormalities.

## Conclusions

Our study evaluated the entire small intestinal mucosal abnormalities of IL by VCE, especially endoscopic complications. Clinical characteristics vary between white villi type and non-white villi type patients with IL. IL has specific VCE abnormalities in addition to classical endoscopic findings.

## Data Availability

The online version includes the data from the current study available at https://doi.org/10.57760/sciencedb.09499.

## Abbreviations

EGD: Esophagogastroduodenoscopy
HAS: Human serum albumin
IL: Intestinal lymphangiectasia
MCT: Medium chain triglycerides
PEG: Polyethylene glycol
PIL: Primary intestinal lymphangiectasia
PLE: Protein-losing enteropathy
SD: Standard deviation
SIL: Secondary intestinal lymphangiectasia
SPSS: Statistical product and service solution
VCE: Video capsule endoscopy
